# Association of Preoperative Neutrophil-to-Lymphocyte Ratio with Postoperative Acute Kidney Injury and Mortality Following Major Noncardiac Surgeries

**DOI:** 10.1007/s00268-022-06878-2

**Published:** 2023-01-21

**Authors:** Jie Wang, Yaodan Bi, Jun Ma, Yijun He, Bin Liu

**Affiliations:** 1 Department of Anesthesiology, West China Hospital of Sichuan University, No. 37 Guoxue Alley, Wuhou District, Chengdu, 610041 Sichuan China; 2 Department of Anesthesiology, Peking Union Medical College Hospital, Chinese Academy of Medical Sciences and Peking Union Medical College, Beijing, China; 3 Department of Medical Imaging, West China Hospital of Sichuan University, Chengdu, Sichuan China

## Abstract

**Background:**

Acute kidney injury (AKI) is a major complication that occurs following an operation. Therefore, there is an increasing need to discover new predictors of AKI. We hypothesized that the preoperative neutrophil-to-lymphocyte ratio (NLR) was associated with postoperative AKI and in-hospital mortality following noncardiac surgery.

**Methods:**

This is a retrospective observational study of patients who underwent noncardiac surgery at Sichuan University West China Hospital from 2018 to 2020. Multivariable logistic regression was performed as the major analytic method. In addition, sensitivity and subgroup analyses were performed to validate the results.

**Results:**

A total of 44,065 patients were included in this study. The prevalence of postoperative AKI was 5.62%, and the in-hospital mortality was 1.58%. Multivariable analysis demonstrated that NLR ≥ 5 was independently associated with the development of postoperative AKI (OR 1.42, 1.24–1.73; *P* < 0.001) and in-hospital mortality (OR 2.03, 1.63–2.52; *P* < 0.001). Similar results were achieved when propensity-score matching was performed for patients with NLR ≥ 5 and < 5 on the baseline. In stratified analysis, the associations remained persistent in most subgroups. For the sensitivity analysis, we took NLR as a continuous variable and demonstrated the potential linear relationship between NLR and postoperative AKI and mortality.

**Conclusions:**

Our results indicated that preoperative NLR is associated with the prevalence of postoperative AKI and in-hospital mortality that occur after major noncardiac surgery. These findings suggest that NLR has the potential to be a significant correlation biomarker associated with perioperative risk assessment of patients undergoing noncardiac surgeries.

## Introduction

Acute kidney injury (AKI) is a major complication reported in patients after an operation. In patients undergoing major surgery, the incidence of AKI is reported to be around 6.1–22.4%. AKI is associated with extended duration of hospital stay, cost, and mortality [1–3]. Development of AKI could contribute to the development of chronic kidney disease (CKD) or end-stage renal disease (ESRD). There is a gradual increase in the incidence of AKI and its impact on long-term health and cost is far greater than previously acknowledged [4]. Therefore, an early and rapid diagnosis and treatment of AKI are crucial in the management of patients with syndromes caused or associated with AKI [4, 5]. Since there are no widely used prediction models for AKI in noncardiac surgery, there is a growing need for the discovery of novel correlation biomarkers of AKI.

Among the existing methods, preoperative determination of certain biomarkers has been increasingly used in the prediction of postoperative adverse outcomes [6, 7]. The neutrophil-to-lymphocyte ratio (NLR) is a commonly used prognostic marker that is indicative of systemic inflammation and resulting immunosuppression. Recent studies have reported an association between that high levels of NLR with severe COVID-19 and mortality [8]. NLR has also been associated with adverse outcomes in malignant tumors including breast, ovary, and lung [9, 10]. AKI could independently contribute to an increase in mortality via impairment of neutrophil function and the patient’s inability to effectively clear the infection [11]. In some cases, elevated levels of neutrophil gelatinase-associated lipocalin (NGAL) could be indicative of renal impairment in the absence of any other indicators of AKI [11–13]. However, there is not enough evidence to suggest the association of preoperative NLR with an increased prevalence of postoperative AKI and in-hospital mortality after noncardiac surgery. In this study, we investigated the relationship of NLR associated with major noncardiac surgery with postoperative AKI and postoperative in-hospital mortality.

## Materials and methods

### Study design and data collection

This study was approved by Ethical Committee on Biomedical Research (Ethical Committee 554), West China Hospital of Sichuan University (Chairperson Prof Deng Shaolin) on May 13, 2021. This is a retrospective observational study, which meets the requirement of “Strengthening observational studies in epidemiological statements”. Original data were retrieved from the Hospital Information System and the Anesthesia Information Management System. Owing to the sensitivity of the data, data collection was performed blindly by the staff of the hospital data processing center. Baseline characteristics of the preoperative assessment forms were compiled into standardized forms by independent investigators. Subsequent data analysis was performed by qualified researchers abiding by the confidentiality agreement for human subjects. All data were anonymously identified and analyzed for confidentiality purposes.

A total of 165,803 patients aged no less than 18 years who underwent elective or emergency surgery at the Information Center of West China Hospital of Sichuan University from February 2018 to November 2020 were included in this study using database analysis. Patients were excluded for the following reasons: (1) they were undergoing cardiac, ophthalmic, obstetric, urologic, or diagnostic surgery; (2) preoperative NLR measurements were not available; (3) serum creatinine levels were not measured before or during one week after surgery; (4) presence of coexisting end-stage renal disease.

The primary outcome of the data analyses was postoperative AKI and in-hospital mortality. AKI was defined by the serum creatinine criteria of the kidney disease: Improving Global Outcomes (KDIGO) criteria classification [14]. Based on the serum creatinine criteria, AKI was indicated by an increase in serum creatinine of ≥ 26.5 mmol/L within 48 h after surgery or ≥ 1.5 times baseline within seven postoperative days. Preoperative concentration was defined as the most recent recorded measurement taken within 14 days before the surgery. NLR was calculated based on recorded absolute total neutrophil and lymphocyte counts (dividing the number of the former by the number of the latter), based on the last preoperative routine blood test. The elevated NLR group was defined as corresponding to NLR ≥ 5 serum levels and the other patients were categorized into the normal group.

### Statistical analysis

The demographics of patients and variables of perioperative tests between the preoperative NLR groups (normal versus elevated NLR) were defined. Continuous data were analyzed using the Student’s *t* test or the Mann–Whitney test for differences between the compared groups. The results were represented in terms of the median and interquartile range (25th–75th percentile) or mean ± SD. Categorical data were compared using the Fisher exact test or *X*^2^ test and presented as numbers (percentages). A *P* value of < 0.05 was considered statistically significant.

We used multivariable logistic regression models to the association of preoperative serum NLR with postoperative AKI while adjusting for potential confounding variables (model 1). LASSO (Least Absolute Shrinkage and Selection Operator) approach was applied to further select variables in a logistic regression model. The data were adjusted for the following variables to understand the effect of NLR on postoperative AKI and in-hospital mortality: sex; age; anesthesia duration; the American Society of Anesthesiologists Physical Status (ASA PS); emergency surgery; general anesthesia; comorbid diseases: hypertension, transplanted organ, liver disease; WBC; Hemoglobin (HB); albumin; *γ*-glutamyl transpeptidase; serum kalium (K); serum magnesium (Mg); surgical subspecialty: central nervous system, general, other; urine output (ml/kg/h); duration of mean arterial pressure (MAP) < 50 mmHg. As part of the sensitivity analysis, NLR was included as a continuous variable for postoperative AKI in multivariable regression analysis (model 2), instead of a dichotomous variable which was bounded by a cutoff point of 5. We used a restricted cubic spline to model potential nonlinear relationships between the risk of outcomes and NLR. The results were represented in terms of odds ratios (ORs) with 95% CIs. A *P* value of < 0.05 was considered statistically significant.

The association between preoperative NLR and postoperative AKI was further investigated using propensity-score (PS) matching to balance measured baseline confounders and risk factors. A logistic regression model was constructed based on roughly the same variables of multivariable logistic regression analysis for calculating the PS for each subject of the two groups, which were categorized based on the preoperative NLR values. PS matching between the two groups (≥ 5 of the NLR group vs. the < 5 of NLR groups) was performed in a one-to-one fashion by calipers of width equal to 0.1 of the standard deviation of the logit of the PS. Covariate balances after matching were validated by comparing the standardized differences [15]. After PS matching, a standardized difference greater than 10% was considered imbalanced. Pearson *χ*^2^ test was performed to compare the prevalence of postoperative AKI between the two groups.

Multivariable logistic regression analysis was further performed to develop the following risk model: risk score = (0.75 × sex) + (1.01 × age) + (1.91 × Anesthesia duration) + (1.64 × ASA PS grades) + (0.67 × emergency surgery) + (0.41 × General anesthesia) + (2.0 × Hypertension) + (3.51 × Transplanted organ) + (1.22 × Liverdisease) + (0.99 × HB) + (0.99 × Albumin) + (1.32 × Albumin) + (0.16 × Mg) + (1.43 × Department) + (0.84 × Urine output) + (1.83 × Duration of MAP < 50 mmHg) + (1.23 × NLR). Receiver operating characteristic plots were used to compute the sensitivity and specificity.

R version 4.1.3 software was used to carry out statistical analyses. In this study, *P* < 0.05 was considered statistically significant and all tests carried out were 2-tailed.

## Results

### Baseline characteristics

**Fig. 1:**
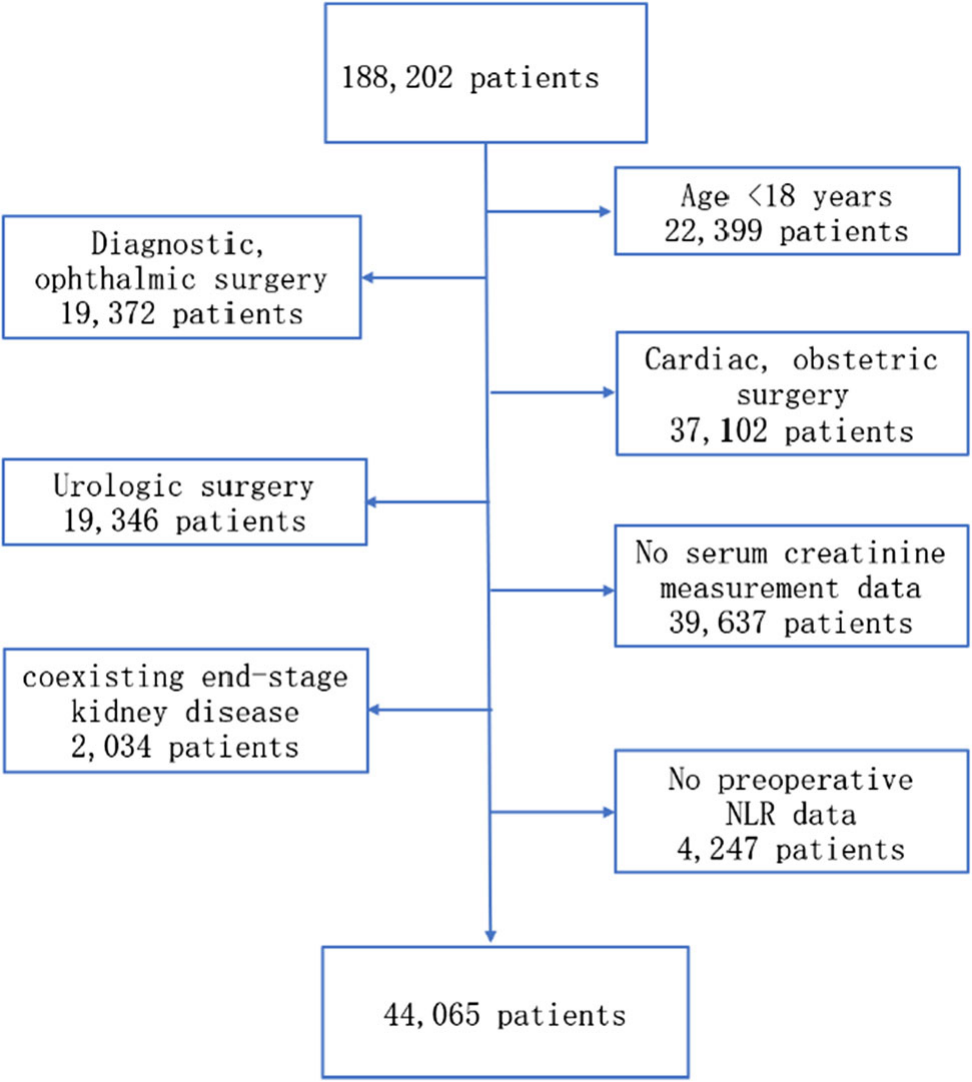
Patient flowchart

A total of 144,137 patients were excluded from a total of 188,202 patients undergoing elective or emergency surgery during the study period for various reasons (Fig. 1). A total of 44, 065 patients were finally included in the study. Baseline patient characteristics, preoperative and perioperative variables were compared between patients with normal NLR and elevated NLR groups and are summarized in Table 1. A total of 6588 patients (14.95%) demonstrated elevated preoperative NLR, while 37,477 (85.05%) exhibited normal levels. In the current study, postoperative AKI was prevalent in 2477 patients (5.62%), including 1826 patients (4.14%) with KDIGO Stage 1, 349 patients (0.79%) with Stage 2, and 302 patients (0.69%) with Stage 3 AKI. A total of 704 patients died following the operation corresponding to a mortality rate of 1.58%.

**Table 1:** Baseline patient characteristics in relation to preoperative NLR

Variable	Overall	NLR < 5	NLR ≥ 5	*P*-value
N	44,065	37,477	6588	
*Sex (%)*				
Male	23,383 (53.1)	19,346 (51.6)	4037 (61.3)	< 0.001
Female	20,682 (46.9)	18,131 (48.4)	2551 (38.7)	
Age (mean (SD))	53.23 (15.35)	53.19 (15.02)	53.46 (17.11)	0.193
BMI (mean (SD))	23.23 (3.68)	23.34 (3.63)	22.60 (3.89)	< 0.001
Surgery duration (median [IQR])	120 [75, 190]	119 [75, 187]	135 [79, 209]	< 0.001
Anesthesia duration (median [IQR])	187 [130, 269]	183 [129, 264]	211 [144, 291]	< 0.001
*General anesthesia (%)*				
NO	674 (1.5)	455 (1.2)	219 (3.3)	< 0.001
YES	43,391 (98.5)	37,022 (98.8)	6369 (96.7)	
*ASA-PS (%)*				
I-II	28,777 (65.3)	26,304 (70.2)	2473 (37.5)	< 0.001
III	14,464 (32.8)	10,958 (29.2)	3506 (53.2)	
IV-V	824 (1.9)	215 (0.6)	609 (9.2)	
*Emergency case (%)*				
Emergency	4018 (9.1)	1247 (3.3)	2771 (42.1)	< 0.001
Elective	40,047 (90.9)	36,230 (96.7)	3817 (57.9)	
Comorbid diseases				
*Ischemic heart disease (%)*				
NO	42,599 (96.7)	36,238 (96.7)	6361 (96.6)	0.585
YES	1466 (3.3)	1239 (3.3)	227 (3.4)	
*Hypertension (%)*				
NO	35,617 (80.8)	30,510 (81.4)	5107 (77.5)	< 0.001
YES	8448 (19.2)	6967 (18.6)	1481 (22.5)	
*Cerebrovascular disease (%)*				
NO	43,730 (99.2)	37,207 (99.3)	6523 (99.0)	0.027
YES	335 (0.8)	270 (0.7)	65 (1.0)	
*Transplanted organ*				
NO	43,917 (99.7)	37,377 (99.7)	6540 (99.3)	< 0.001
YES	148 (0.3)	100 (0.3)	48 (0.7)	
*Diabetes (%)*				
NO	40,249 (91.3)	34,388 (91.8)	5861 (89.0)	< 0.001
YES	3816 (8.7)	3089 (8.2)	727 (11.0)	
*Liver disease (%)*				
NO	33,671 (76.4)	28,343 (75.6)	5328 (80.9)	< 0.001
YES	10,394 (23.6)	9134 (24.4)	1260 (19.1)	
*Surgical subspecialty (%)*				
CNS	4223 (9.6)	3230 (8.6)	993 (15.1)	< 0.001
General (%)	9711 (22.0)	7789 (20.8)	1922 (29.2)	
Orthopedics (%)	13,390 (30.4)	11,324 (30.2)	2066 (31.4)	
Other	16,741 (38.0)	15,134 (40.4)	1607 (24.4)	
*Duration of MAP* < *50 mmHg (%)*				
0 min	41,786 (94.8)	35,701 (95.3)	6085 (92.4)	< 0.001
1-30 min	2206 (5.0)	1722 (4.6)	484 (7.3)	
> 30 min	73 (0.2)	54 (0.1)	19 (0.3)	
*Intraoperative fluid volume*				
Colloid (median [IQR])	500 [0, 550]	500 [0, 500]	500 [0, 1000]	< 0.001
Crystalloid (median [IQR])	1100 [700, 1700]	1100 [700, 1700]	1400 [900, 2100]	< 0.001
Urine output (median [IQR])	2.87 [1.38, 4.78]	2.94 [1.40, 4.82]	2.52 [1.31, 4.52]	< 0.001
*Preoperative laboratory tests*				
HB (mean (SD))	130.64 (21.20)	132.78 (19.49)	118.49 (25.90)	< 0.001
PLT (mean (SD))	196.70 (82.02)	194.36 (75.96)	209.97 (109.38)	< 0.001
WBC (median [IQR])	5.85 [4.77, 7.34]	5.59 [4.64, 6.73]	9.47 [7.16, 12.73]	< 0.001
ALT (mean (SD))	28.69 (57.53)	27.23 (35.57)	36.99 (121.89)	< 0.001
AST (median [IQR])	21 [17, 27]	20.00 [17, 26]	22.00 [17, 34]	< 0.001
AKP (median [IQR])	78 [63, 98]	77 [63, 96]	80 [63, 107]	< 0.001
γ-GT (median [IQR])	22 [14, 42]	22 [14, 39]	27 [16, 60]	< 0.001
TP (mean (SD)	68.34 (6.69)	68.91 (6.08)	65.08 (8.74)	< 0.001
Albumin (mean (SD))	42.56 (5.00)	43.23 (4.37)	38.77 (6.46)	< 0.001
BUN (median [IQR])	4.9 [4.0, 6.1]	4.9 [4.0, 6.0]	5.2 [3.9, 7.0]	< 0.001
Creatinine (median [IQR])	67 [56, 79]	67 [57, 79]	66 [53, 81]	< 0.001
GFR (mean (SD))	96.76 (21.39)	96.80 (19.95)	96.53 (28.19)	0.356
Uric acid (mean (SD))	311.07 (97.98)	317.35 (91.99)	275.32 (120.71)	< 0.001
TC (median [IQR])	1.22 [0.88, 1.74]	1.24 [0.90, 1.76]	1.12 [0.81, 1.58]	< 0.001
Cholesterol (mean (SD))	4.39 (1.10)	4.49 (1.05)	3.86 (1.24)	< 0.001
CK (median [IQR])	75 [54, 108]	75 [55, 104]	75.5 [43, 160]	0.023
LDH (median [IQR])	165 [143, 193]	162 [142, 187]	189 [157, 243]	< 0.001
HBDH (median [IQR])	128 [112, 150]	126.0 [111, 146]	147[122, 189]	< 0.001
Na (mean (SD))	140.78 (2.95)	141.15 (2.44)	138.68 (4.39)	< 0.001
K (mean (SD))	4.04 (0.38)	4.05 (0.36)	3.95 (0.49)	< 0.001
Ca (mean (SD))	2.26 (0.13)	2.28 (0.11)	2.17 (0.17)	< 0.001
Hospital days (median [IQR])	9 [6, 13]	8 [6, 12]	11 [7, 18]	< 0.001
*Postoperative AKI (%)*				
YES	2477 (5.6)	1653 (4.4)	824 (12.5)	< 0.001
NO	41,588 (94.4)	35,824(95.4)	5764 (87.5)	
*Death (%)*				
YES	704 (1.6)	251 (0.7)	453 (6.9)	< 0.001
NO	43,361 (98.4)	37,225 (99.3)	6135 (93.1)	

*NLR* Neutrophil-to-lymphocyte ratio, *BMI* Body mass index, *ASA PS* American Society of Anesthesiologists physical status, *IQR* Interquartile range, *CI* confidence interval, *CNS* Central nervous system, *MAP* Mean arterial pressure, *HB* Hemoglobin, *PLT* Platelet, *WBC* White blood cells, *ALT* Alanine transaminase, *AST* Aspartate transaminase, *AKP* Alkaline phosphatase *γ-GT,γ* Glutamyl transpeptidase, *TP* Total protein, *BUN* Blood urea nitrogen, *GFR* Glomerular filtration rate, *TC* Triglyceride; *CK* Creatine kinase, *LDH* Lactic dehydrogenase, *HBDH α*- Hydroxybutyrate dehydrogenase, *AKI* Acute Kidney Injury

According to the baseline patient characteristics (Table 1), the proportion of male patients was significantly higher (*P* < 0.001) in the ≥ 5 NLR group than that in the < 5 NLR group. BMI was found to be significantly lower (*P* < 0.001) in patients with ≥ 5 of NLR. Furthermore, patients with elevated NLR had higher ASA PS grades, and a higher prevalence of emergency surgery, central nervous system surgery, hypertension, diabetes, and cerebrovascular disease (*P* < 0.05). The duration of mean arterial pressure < 50 mmHg was significantly longer in the NLR ≥ 5 group (*P* < 0.001). Additionally, there was significantly more colloid and crystalloid infusion in the NLR ≥ 5 group (*P* < 0.001), but the urine output was significantly lower in the NLR ≥ 5 group (*P* < 0.001) than in the NLR < 5 group. Moreover, the NLR ≥ 5 group had longer hospital days and a higher proportion of unplanned ICU admissions than the NLR < 5 groups (*P* < 0.001).

### NLR and postoperative outcomes

The crude AKI rate was found to be 4.94% (1853 patients) for patients with normal NLR compared to that of 9.47% (624 patients) in patients with elevated NLR (OR 2.61, 2.32- 2.94; *P* < 0.001). Similarly, crude mortality was found to be 0.7% (251 patients) in patients with normal NLR, which was significantly lower than that in the elevated NLR group (6.9%, 453 patients; OR 10.95, 9.37–12.82; *P* < 0.001). A total of 21 variables were included in the model for multivariable logistic regression analysis (Table 2). NLR ≥ 5 was independently associated with the development of postoperative AKI (OR 1.42, 1.24- 1.73; *P* < 0.001) and mortality (OR 2.03, 1.63- 2.52; *P* < 0.001) after adjusting for potential confounding factors (sex; age; anesthesia duration; ASA PS; emergency surgery; general anesthesia; comorbid diseases: hypertension, transplanted organ, liver disease; *γ*-glutamyl transpeptidase; surgical subspecialty; urine output (ml/kg/h); duration of MAP < 50 mmHg; Hemoglobin (HB); albumin) (Table 2, model 1).

**Table 2:** Multivariable logistic regression model for association between preoperative NLR and postoperative acute kidney injury

	AKI				Death			
	Unadjusted		lrm-adjusted		Unadjusted		lrm-adjusted	
Model 1, primary exposure	Odds ratio 95% CI	*P* value	Odds ratio 95% CI	*P* value	Odds ratio 95% CI	*P* value	Odds ratio 95% CI	*P* value
Female	0.62 [0.55, 0.69]	< 0.001	0.75 [0.66, 0.85]	< 0.001	0.59 [0.50, 0.69]	< 0.001	0.80 [0.67, 0.96]	0.018
Age	1.03 [1.02, 1.03]	< 0.001	1.01 [1.01, 1.02]	< 0.001	1.02 [1.01, 1.02]	< 0.001	1.01 [1.00, 1.01]	0.005
Anesthesia duration*	2.69 [2.40, 3.00]	< 0.001	1.92 [1.70, 2.18]	< 0.001	1.71 [1.47, 1.99]	< 0.001	1.06 [0.90, 1.25]	0.496
ASA PS**	3.18 [2.92, 3.46]	< 0.001	1.64 [1.47, 1.84]	< 0.001	10.99 [9.69, 12.48]	< 0.001	3.07 [2.64, 3.57]	< 0.001
Elective surgery	0.27 [0.24, 0.31]	< 0.001	0.67 [0.56, 0.80]	< 0.001	0.06 [0.05, 0.07]	< 0.001	0.35 [0.28, 0.44]	< 0.001
General anesthesia	0.41 [0.31, 0.57]	< 0.001	0.41 [0.29, 0.57]	< 0.001	0.35 [0.24, 0.53]	< 0.001	0.43 [0.28, 0.68]	< 0.001
*Comorbid diseases*								
Ischemic heart disease	1.93 [1.53, 2.41]	< 0.001	0.98 [0.76, 1.24]	0.877	1.92 [1.38, 2.59]	< 0.001	1.15 [0.79, 1.64]	0.452
Hypertension	2.45 [2.19, 2.74]	< 0.001	1.99 [1.75, 2.27]	< 0.001	1.69 [1.43, 1.99]	< 0.001	1.00 [0.82, 1.23]	0.981
Transplanted organ	15.29[10.71,21.53]	< 0.001	3.55 [2.35, 5.31]	< 0.001	7.62 [4.34, 12.47]	< 0.001	1.27 [0.64, 2.38]	0.477
Liver disease	1.64 [1.46, 1.84]	< 0.001	1.22 [1.08, 1.39]	0.002	1.47 [1.25, 1.73]	< 0.001	1.61 [1.32, 1.96]	< 0.001
Diabetes	2.04 [1.76, 2.36]	< 0.001	1.01 [0.86, 1.19]	0.86	2.32 [1.90, 2.81]	< 0.001	1.28 [1.01, 1.61]	0.039
WBC*	1.61 [1.41, 1.84]	< 0.001	0.91 [0.79, 1.05]	0.191	6.95 [5.99, 8.06]	< 0.001	1.21 [1.01, 1.45]	0.038
HB	0.98 [0.98, 0.98]	< 0.001	0.99 [0.99, 0.99]	< 0.001	0.97 [0.97, 0.97]	< 0.001	0.99 [0.99, 1.00]	0.001
Albumin	0.91[0.90, 0.91]	< 0.001	0.99 [0.97, 1.00]	0.035	0.83 [0.82, 0.84]	< 0.001	0.94 [0.92, 0.95]	< 0.001
γ-GT*	2.00 [1.78, 2.25]	< 0.001	1.33 [1.17, 1.51]	< 0.001	2.41 [2.06, 2.80]	< 0.001	1.48 [1.22, 1.80]	< 0.001
K	0.74 [0.64, 0.85]	< 0.001	1.02 [0.90, 1.16]	0.734	0.56 [0.46, 0.68]	< 0.001	1.04 [0.88, 1.23]	0.621
Mg	0.02 [0.01, 0.04]	< 0.001	0.16 [0.09, 0.29]	< 0.001	0.02 [0.01, 0.05]	< 0.001	1.89 [0.92, 3.88]	0.084
Department								
Orthopedic surgery	OR = 1		OR = 1		OR = 1		OR = 1	
CNS	1.04 [0.86, 1.24]	0.692	1.42 [1.13, 1.79]	0.003	4.32 [3.66, 5.08]	< 0.001	6.60 [4.82, 9.15]	< 0.001
General	1.38 [1.22, 1.55]	< 0.001	1.45 [1.21, 1.74]	< 0.001	1.37 [1.16, 1.62]	< 0.001	1.53 [1.12, 2.12]	0.008
Other	1.44 [1.29, 1.60]	< 0.001	1.83 [1.56, 2.16]	< 0.001	0.79 [0.68, 0.93]	0.004	2.05 [1.52, 2.81]	< 0.001
Urine output*	0.58 [0.52, 0.63]	< 0.001	0.85 [0.75, 0.96]	0.008	0.70 [0.61, 0.80]	< 0.001	0.84 [0.72, 0.98]	0.03
Duration of MAP < 50 mmHg**	3.06 [2.65, 3.53]	< 0.001	1.90 [1.61, 2.22]	< 0.001	4.28 [3.60, 5.05]	< 0.001	2.24 [1.80, 2.78]	< 0.001
NLR ≥ 5	2.61 [2.32, 2.94]	< 0.001	1.42 [1.24, 1.73]	< 0.001	10.95 [9.37, 12.82]	< 0.001	2.03 [1.63, 2.52]	< 0.001
*Model 2, secondary exposure*								
NLR*	1.88 [1.76, 2.00]	< 0.001	1.30 [1.18, 1.44]	< 0.001	3.84 [3.55, 4.15]	< 0.001	1.58 [1.39, 1.78]	< 0.001

Model 2 adjusts for same variables as model 1

*ASA PS* American Society of Anesthesiologists physical status, *CI* Confidence interval, *WBC* White blood cells, *γ-GT*,*γ* Glutamyl transpeptidase, *CNS* Central nervous system, *MAP* mean arterial pressure, *NLR* Neutrophil-to-lymphocyte ratio

^*^The variables were analyzed after logarithmic transformation. **The variables were analyzed in terms of grade data

ASA-PS: I is a healthy patient; II is mild systemic disease but no functional limitations; III is severe systemic disease with definite functional limitations; IV is severe systemic disease that is a constant threat to life; and V is a moribund patient unlikely to survive 24 h with or without an operation. Duration of MAP < 50 mmHg: 0, 0 min; 1, 1-30 min; 2, > 30 min; HB: Hemoglobin

Patient characteristics and intraoperative variables after PS matching on NLR are summarized in Table 3. All variables achieved standardized differences not greater than 10%, which implied efficient balancing of the two groups. The prevalence of postoperative AKI was significantly higher in patients with ≥ 5 of NLR (8.81%, 380 patients; OR = 1.71, 95% CI, 1.57–2.29; *P* < 0.001) as compared to patients with < 5 of NLR (4.92%, 212 patients), following PS matching. Similarly, postoperative in-hospital mortality was significantly greater in patients with ≥ 5 of NLR (206 patients, 4.8%) than that in patients with < 5 of NLR (2.6%, 114 patients; OR = 1.80, 1.39–2.33; *P* < 0.001). These findings were consistent with the results obtained before propensity-score matching.

**Table 3:** Propensity-score matching of patient characteristics and intraoperative variables

Variable	Overall	NLR < 5	NLR ≥ 5	*P*-value
*n*	8630	4315	4315	
*sex (%)*				
Male	5273 ( 61.1)	2642 ( 61.2)	2631 ( 61.0)	0.825
Female	3357 ( 38.9)	1673 ( 38.8)	1684 ( 39.0)	
age (mean (SD))	54.07 (16.76)	54.20 (16.67)	53.94 (16.86)	0.481
BMI (mean (SD))	22.93 (3.85)	23.12 (3.85)	22.84 (3.80)	0.048
Surgery duration (median [IQR])	132 [78, 210]	130 [79, 210]	134 [76, 206]	0.519
Anesthesia duration (median [IQR])	205 [137, 289]	203 [137, 290]	206 [138, 288]	0.882
*General anesthesia (%)*				
NO	269 (3.1)	147 (3.4)	122 (2.8)	0.137
YES	8361 ( 96.9)	4168 ( 96.6)	4193 ( 97.2)	
*ASA-PS (%)*				
I-II	3908 ( 45.3)	1955 ( 45.3)	1953 ( 45.3)	< 0.001
III	4327 ( 50.1)	2211 ( 51.2)	2116 ( 49.0)	
IV-V	395 ( 4.6)	149 ( 3.5)	246 ( 5.7)	
Emergency case (%)				
Emergency	1858 ( 21.5)	885 ( 20.5)	973 ( 22.5)	0.023
Elective	6772 ( 78.5)	3430 ( 79.5)	3342 ( 77.5)	
*Comorbid diseases*				
*Ischemic heart disease (%)*				
NO	8342 ( 96.7)	4177 ( 96.8)	4165 ( 96.5)	0.51
YES	288 ( 3.3)	138 ( 3.2)	150 ( 3.5)	
*Hypertension (%)*				
NO	6577 ( 76.2)	3266 ( 75.7)	3311 ( 76.7)	0.266
YES	2053 ( 23.8)	1049 ( 24.3)	1004 ( 23.3)	
*Cerebrovascular disease (%)*				
NO	8541 ( 99.0)	4270 ( 99.0)	4271 ( 99.0)	0.827
YES	89 ( 1.0)	45 (1.0)	44 (1.0)	
*Transplanted organ*				
NO	8569 ( 99.3)	4291 ( 99.4)	4278 ( 99.1)	0.123
YES	61 ( 0.7)	24 ( 0.6)	37 (0.9)	
*Diabetes (%)*				
NO	7678 ( 89.0)	3822 ( 88.6)	3856 ( 89.4)	0.257
YES	952 ( 11.0)	493 ( 11.4)	459 ( 10.6)	
*Liver disease (%)*				
NO	6874 ( 79.7)	3440 ( 79.7)	3434 ( 79.6)	0.894
YES	1756 ( 20.3)	875 ( 20.3)	881 ( 20.4)	
*Surgical subspecialty (%)*				
CNS	1084 ( 12.6)	519 ( 12.0)	565 ( 13.1)	0.521
General	2162 ( 25.1)	1072 ( 24.8)	1090 ( 25.3)	
Orthopedics	3157 ( 36.6)	1624 ( 37.6)	1533 ( 35.5)	
Other	2227 ( 25.8)	15,134 (40.4)	1607 (24.4)	
*Duration of MAP* < *50 mmHg (%)*				
0 min	7994 (92.6)	3996 (92.6)	3998 ( 92.7)	0.973
1-30 min	611 (7.1)	307 (7.1)	304 (7.0)	
> 30 min	25 (0.3)	12 (0.3)	13 (0.3)	
*Intraoperative fluid volume*				
Colloid (median [IQR])	500 [0, 1000]	500 [0, 1000]	500 [0, 1000]	0.211
Crystalloid (median [IQR])	1300 [800, 2000]	1300 [800, 2000]	1300 [800, 2000]	0.227
Urine output (median [IQR]	2.68 [1.36, 4.69]	2.76 [1.40, 4.63]	2.61 [1.32, 4.76]	0.447
*Preoperative laboratory tests*				
HB (mean (SD))	123.07 (24.53)	126.92 (23.34)	119.22 (25.08)	< 0.001
PLT (mean (SD))	217.48 (104.44)	230.41 (102.52)	204.55 (104.77)	< 0.001
WBC (median [IQR])	7.85 [6.27, 9.75]	7.69 [6.27, 9.38]	8.06 [6.28, 10.26]	< 0.001
ALT (mean (SD))	34.10 (99.69)	31.34 (42.18)	36.86 (104.48)	0.01
AST (median [IQR])	21 [17, 31]	20 [16, 29]	22 [17, 33]	< 0.001
AKP (median [IQR])	81 [65, 106]	82 [65, 105]	81 [64, 108]	0.822
*γ*-GT (median [IQR])	27.00 [16.00, 56.00]	28.00 [17.00, 55.00]	27.00 [16.00, 59.00]	0.745
TP (mean (SD))	66.36 (7.89)	66.93 (7.44)	65.79 (8.28)	< 0.001
Albumin (mean (SD))	39.94 (5.83)	40.10 (5.47)	39.79 (6.17)	0.014
BUN (median [IQR])	5.1 [3.9, 6.5]	5.0 [3.9, 6.3]	5.2 [3.9, 6.8]	< 0.001
Creatinine (median [IQR])	66 [55, 79]	67[56, 80]	65 [53, 79]	0.003
GFR (mean (SD))	96.68 (26.04)	96.96 (24.31)	96.41 (27.66)	0.329
Uric acid (mean (SD))	291.24 (113.31)	305.50 (107.96)	276.98 (116.71)	< 0.001
TC (median [IQR])	1.21 [0.88, 1.72]	1.29 [0.95, 1.84]	1.12 [0.82, 1.59]	< 0.001
Cholesterol (mean (SD))	4.14 (1.24)	4.28 (1.24)	3.99 (1.23)	< 0.001
CK (median [IQR])	71 [45, 119]	69 [46, 109]	73 [43, 112]	0.023
LDH (median [IQR])	177 [149, 218]	170 [145, 204]	186 [156, 235]	< 0.001
HBDH (median [IQR])	138 [116, 171]	131[113, 159]	145 [121, 184]	< 0.001
Na (mean (SD))	139.52 (3.74)	139.99 (3.28)	139.05 (4.09)	< 0.001
K (mean (SD))	3.99 (0.45)	4.00 (0.42)	3.99 (0.48)	0.239
Ca (mean (SD))	2.21 (0.15)	2.23 (0.14)	2.20 (0.16)	0.032
Hospital days (median [IQR])	10 [7, 16]	10 [7, 15]	11 [7, 18]	< 0.001
*Postoperative AKI (%)*				
YES	592 (6.8)	212 (4.9)	380 (8.8)	< 0.001
NO	8038 (93.2)	4103(95.1)	3935(91.2)	
*Death (%)*				
YES	320 (3.7)	114 (2.6)	206 (4.8)	< 0.001
NO	8310 (96.3)	4201 (97.4)	4109 (95.2)	

*NLR* neutrophil-to-lymphocyte ratio, *BMI* Body mass index, *ASA PS* American Society of Anesthesiologists physical status, *IQR* interquartile range, *CI* confidence interval, *CNS* central nervous system, *MAP* mean arterial pressure, *HB* Hemoglobin, *PLT* platelet, *WBC* White blood cells, ALT Alanine transaminase, *AST* Aspartate transaminase, *AKP* Alkaline phosphatase, *γ-GT, γ*-Glutamyl transpeptidase, *TP* Total protein, *BUN* Blood urea nitrogen, *GFR* Glomerular filtration rate, *TC* Triglyceride *CK* Creatine kinase, *LDH* Lactic dehydrogenase, *HBDH α*-Hydroxybutyrate dehydrogenase, *AKI* Acute Kidney Injury

### Subgroup analysis

Subgroup analyses were performed based on ASA physical status, surgical urgency, type of anesthesia, surgical site, as well as hypertension, diabetes, intraoperative urine output, and preoperative GFR, which are factors previously reported to be associated with AKI. After adjustment for all potential confounders listed in Table 2 by multivariable logistic regression, elevated levels of preoperative NLR remained independent and significantly associated with increased incidences of postoperative AKI and mortality in most subgroups. With stratification by IV-V ASA PS, cerebrovascular disease, ischemic heart disease and diabetes existed subgroups, the risk of AKI was higher in the NLR ≥ 5 group than that in the normal NLR group, however, the difference failed to achieve statistical significance (Table 4).

**Table 4:** OR (95% CI) for the association between elevated NLR and postoperative AKI and death in subgroups

AKI	NLR < 5	NLR ≥ 5	*P* value
*ASA*			
I-II (*n* = 28,777)	Reference	1.28 [1.03, 1.59]	< 0.001
III (*n* = 14,464)	Reference	1.59 [1.35, 1.94]	< 0.001
IV-V (*n* = 824)	Reference	1.11 [0.98, 1.63]	0.213
*Emergency case*			
Emergency (*n* = 4018)	Reference	1.33 [1.13, 1.71]	< 0.001
Elective (*n* = 40,047)	Reference	1.47 [1.21, 1.80]	< 0.001
*General anesthesia*			
Yes (*n* = 43,391)	Reference	1.48 [1.26, 1.79]	< 0.001
No (*n* = 674)	Reference	1.13 [1.01, 1.67]	0.013
*Hypertension*			
NO (*n* = 35,617)	Reference	1.41 [1.13–1.65]	< 0.001
YES (*n* = 8448)	Reference	1.49 [1.12–1.81]	0.032
*Cerebrovascular disease*			
NO (*n* = 43,730)	Reference	1.45[1.13–1.76]	< 0.001
YES (*n* = 335)	Reference	0.75 [0.27–1.81]	0.243
*Ischemic heart disease*			
NO (*n* = 42,599)	Reference	1.41 [1.13–1.74]	< 0.001
YES (*n* = 1466)	Reference	1.31 [0.87–2.79]	0.354
*Intraoperative urine output*			
< 1 ml/h/kg (n = 7177)	Reference	1.15 [0.98–1.69]	0.074
> = 1 ml/h/kg (n = 36,888)	Reference	1.36 [1.22–1.71]	< 0.001
*Department*			
Orthopedic surgery (*n* = 13,390)	Reference	1.13 [1.02–1.76]	0.034
CNS (*n* = 4223)	Reference	1.42 [0.78–2.32]	0.473
General (*n* = 9711)	Reference	1.77 [1.29–2.37]	< 0.001
Other (*n* = 16,741)	Reference	1.21 [0.95–1.57]	0.167
*Preoperative GFR*			
< 80% (*n* = 7175)	Reference	1.55 [1.20–2.08]	0.001
> = 80% (*n* = 36,890)	Reference	1.22 [1.01–1.51]	0.041
*Diabetes*			
NO (*n* = 40,249)	Reference	1.59 [1.21–1.86]	< 0.001
YES (*n* = 3816)	Reference	1.21 [0.85–1.92]	0.306
*WBC*			
< 10*10^9^/L (*n* = 40,190)	Reference	1.38 [1.03–1.72]	< 0.001
> 10 *10^9^/L(*n* = 3875)	Reference	1.12 [0.81–1.89]	0.726

Death	NLR < 5	NLR ≥ 5	*P* value
*ASA PS*			
I-II (*n* = 28,777)	Reference	1.59 [1.30, 2.95]	< 0.001
III (*n* = 14,464)	Reference	3.01 [2.24, 4.32]	< 0.001
IV-V (*n* = 824)	Reference	1.53 [1.16, 2.57]	0.019
*Emergency case*			
Elective (*n* = 40,047)	Reference	3.05 [2.21, 4.72]	< 0.001
Emergency (*n* = 4018)	Reference	1.59 [1.05, 2.37]	0.003
*General anesthesia*			
Yes (*n* = 43,391)	Reference	2.75 [1.83, 3.36]	< 0.001
No (*n* = 674)	Reference	1.72 [1.51, 4.71]	< 0.001
*Hypertension*			
NO (*n* = 35,617)	Reference	2.43 [1.92–3.25]	< 0.001
YES (*n* = 8448)	Reference	2.81 [1.9–4.16]	< 0.001
*Cerebrovascular disease*			
NO (*n* = 43,730)	Reference	2.58 [1.91–3.16]	< 0.001
YES (*n* = 335)	Reference	1.75 [0.81–11.02]	0.823
*Ischemic heart disease*			
NO (*n* = 42,599)	Reference	2.61 [1.98–3.35]	< 0.001
YES (*n* = 1466)	Reference	2.05 [0.85–4.21]	0.232
*Intraoperative urine output*			
< 1 ml/h/kg (*n* = 7177)	Reference	2.02 [1.19–3.51]	0.025
> = 1 ml/h/kg (*n* = 36,888)	Reference	2.58 [2.01–3.72]	< 0.001
*Department*			
Orthopedic surgery (*n* = 13,390)	Reference	3.18 [2.45–5.33]	< 0.001
CNS (*n* = 4223)	Reference	2.51 [2.07–4.15]	< 0.001
General (*n* = 9711)	Reference	1.32 [0.89–2.12]	0.078
Other (*n* = 16,741)	Reference	2.58 [1.83–4.54]	< 0.001
*Preoperative GFR*			
< 80% (*n* = 7175)	Reference	2.01 [1.35–3.21]	< 0.001
> = 80% (*n* = 36,890)	Reference	2.81 [2.01–3.78]	< 0.001
*Diabetes*			
NO (*n* = 40,249)	Reference	2.37 [1.82–3.13]	< 0.001
YES (*n* = 3816)	Reference	2.23 [1.56–4.16]	< 0.001
*WBC*			
< 10*10^9^/L (*n* = 40,190)	Reference	2.71 [2.01–3.79]	< 0.001
> = 10*10^9^/L(*n* = 3875)	Reference	1.18 [0.81–1.73]	0.307

*CI* Confidence interval, *OR* Odds ratios, *ASA-PS* American society of Anesthesiologists physical status, *CNS* Central nervous system

### Construction of a risk model based on NLR for predicting the risk of postoperative outcomes

**Fig. 2:**
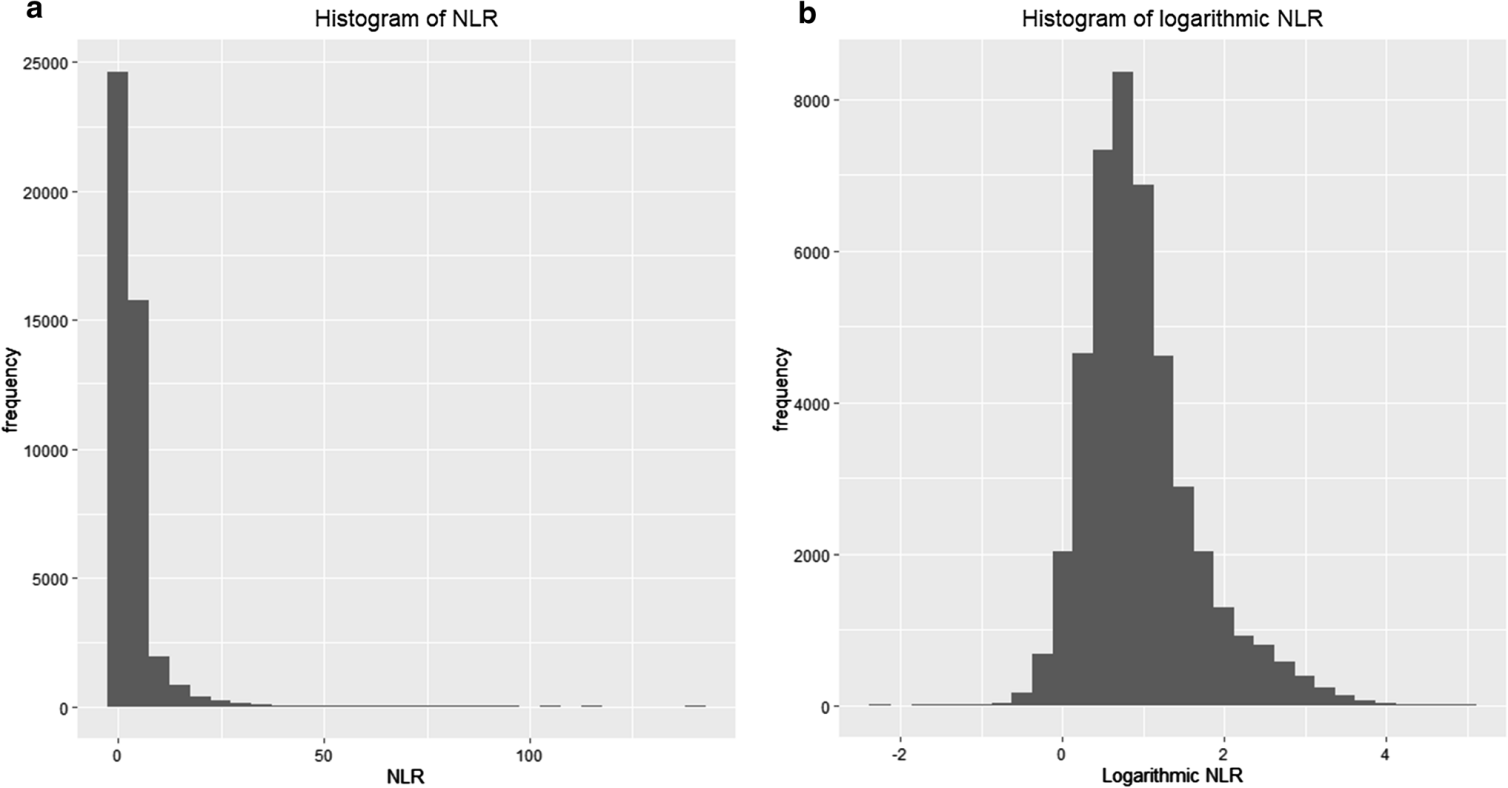
NLR distribution (**a**) and logarithmic transformed NLR distribution (**b**)

**Fig. 3:**
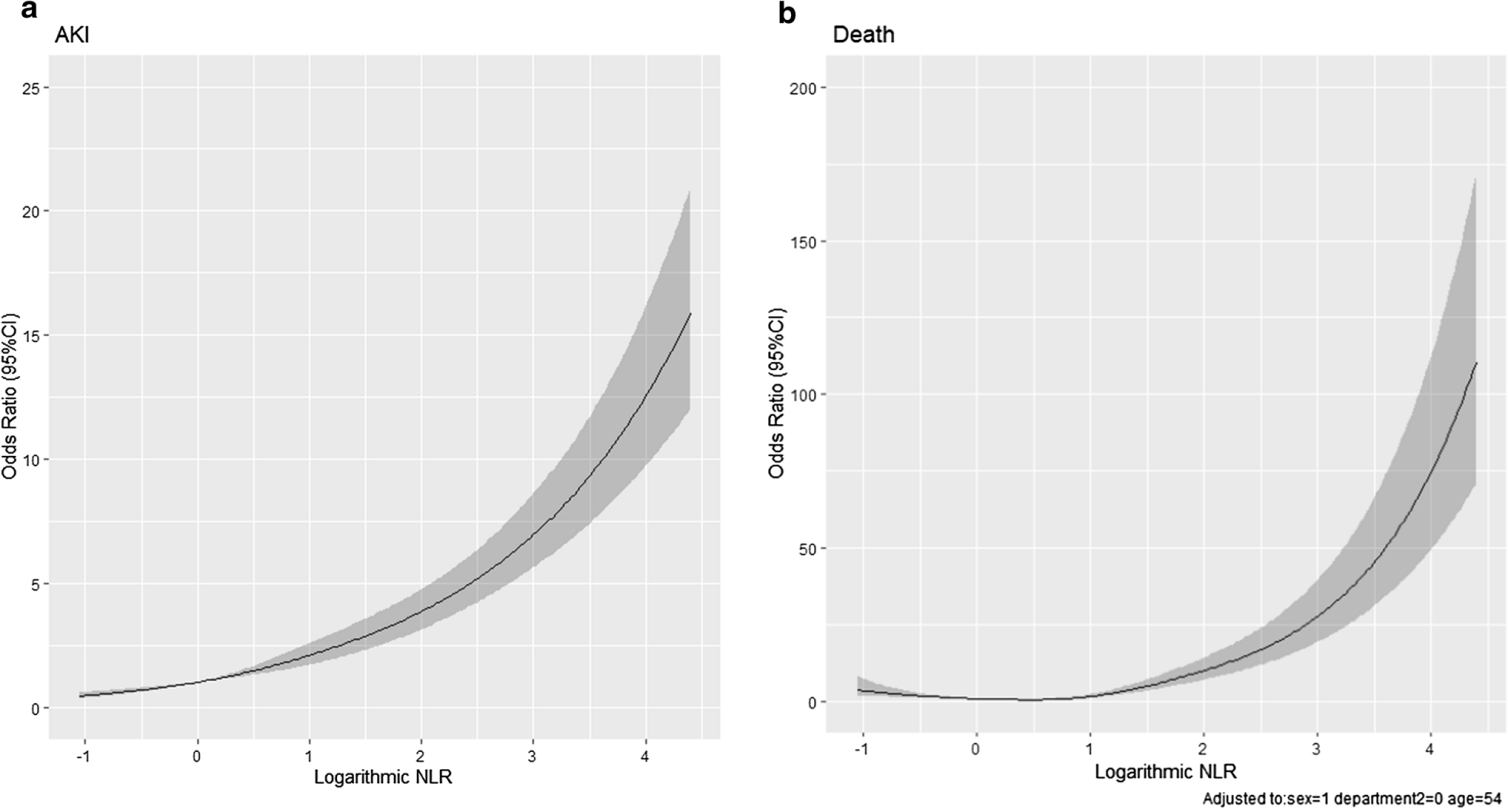
The restricted cubic spline of postoperative AKI (**a**) and Death (**b**) after noncardiac surgery, according to preoperative NLR levels. AKI, acute kidney injury; NLR, neutrophil-to-lymphocyte ratio.

The serum NLR concentration, which was originally a skewed distribution, conformed to normal distribution after logarithmic transformation (Fig. 2). After applying the base-2 logarithm, the multivariable regression analysis produced similar results. As demonstrated by outcomes of Model 2 summarized in Table 2, preoperative serum NLR levels were associated with postoperative AKI (OR 1.30, 1.18–1.44; *P* < 0.001) and in-hospital mortality (OR 1.58, 1.39–1.78; *P* < 0.001). As shown in Fig. 3, the probability of postoperative AKI increased almost linearly in the logarithmic NLR level. There was a corresponding increase in mortality as well.

**Fig. 4:**
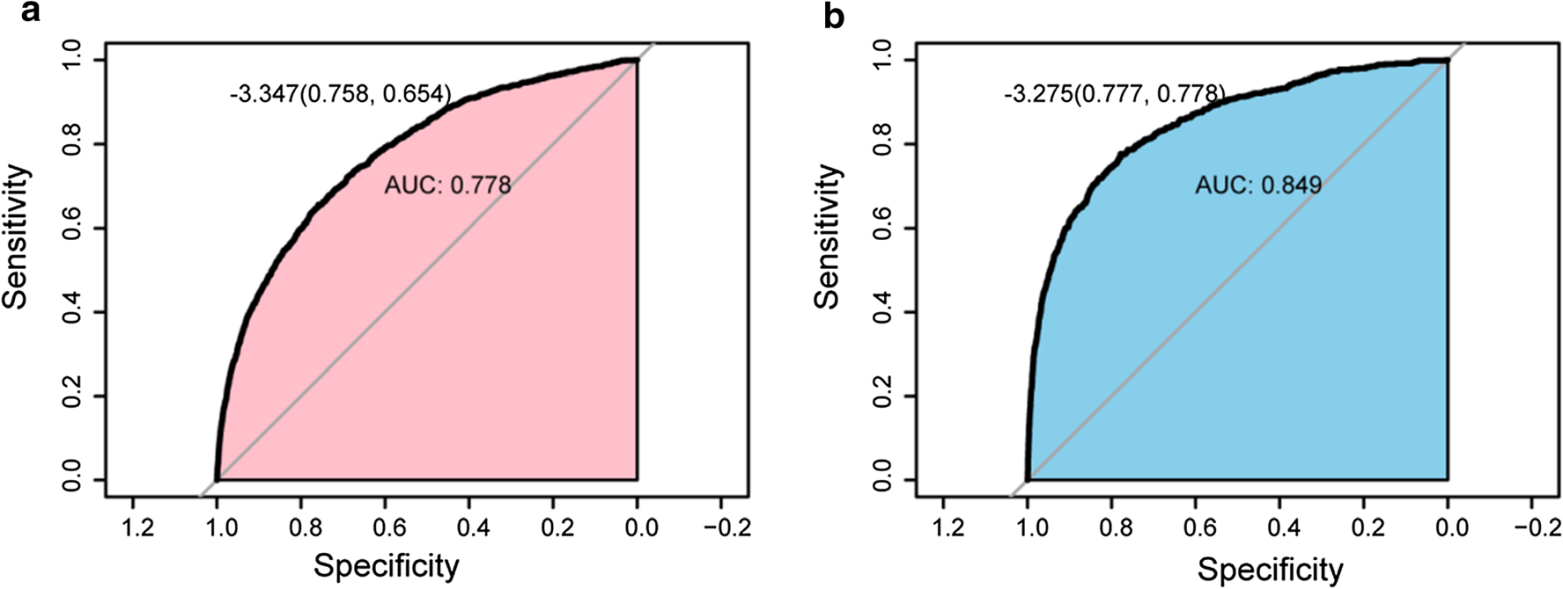
Receiver operating characteristic curves for risk score associated with postoperative AKI (**a**), and Death (**b**) after noncardiac surgery. AKI, acute kidney injury

Finally, a risk model was constructed based on the outcomes of multivariable regression, which contained NLR. As illustrated in Fig. 4, the area under the receiver operating characteristic (AUROC) of the risk score in predicting postoperative AKI was 0.778, the specificity was estimated to be 0.758, with a corresponding sensitivity of 0.654. The AUROC of the risk score in predicting postoperative mortality was 0.849, with a specificity of 0.777, and a sensitivity of 0.778.

## Discussion

A retrospective database analysis was performed on a total of 44,065 surgical cases to understand the relationship between preoperative NLR and postoperative AKI and in-hospital mortality in cases of major surgeries apart from cardiac surgeries. The incident rate of AKI was found to be 5.62% in noncardiac surgical cases estimated based on the serum creatinine criteria of the KDIGO classification. NLR values were categorized into normal and elevated groups based on a cutoff value of 5. Outcomes of multivariable regression analysis and PS matching indicated that NLR ≥ 5 was significantly associated with the development of postoperative AKI and in-hospital mortality.

Furthermore, the findings of this study demonstrated that elevated NLR levels could contribute to a steadily increased risk of adverse events in most subgroup analyses. In the ASA PS IV-V subgroup, elevated NLR was not significantly associated with increased prevalence of postoperative AKI (OR 1.11, 0.98–1.63; *P* = 0.213). This could be due to the presence of existing elevated NLR levels in critically ill patients before the surgery. In addition, we constructed a risk model based on the outcomes of multivariable regression analysis, which included NLR, moreover, the risk score established by predictive factors exhibited excellent accuracy (AUC of AKI = 0.778; AUC of death = 0.849).

The findings of our study suggest that preoperative NLR can be independently associated with the development of postoperative AKI and in-hospital mortality after noncardiac surgery.

The prevalence of AKI in our study was found to be slightly lower than previously reported. This could be attributed to the exclusion of patients with cardiac surgery, urology surgery, and preoperatively existing end-stage renal disease, comprising the high-risk groups for postoperative kidney injury. The majority of excluded patients had no creatinine measurement.

AKI is the most common urologic complication among the perioperative complications of noncardiac surgeries and is independently associated with postoperative mortality. Our findings suggest that NLR could be a contributing risk factor responsible for the increased rate of AKI in noncardiac surgeries. Moreover, the occurrence of AKI may have a certain correlation between preoperative NLR and postoperative mortality. The routine diagnosis of AKI includes an estimation of clinical manifestations such as urine output and creatinine. However, this approach is time-consuming [16], causing a significant delay in the diagnosis and treatment. Nearly 50% of GFR must be lost in a healthy person before a change in serum creatinine is clinically detectable [17, 18]. Unlike routine biomarkers that have been reported to predict kidney injury such as urine interleukin-18 and plasma neutrophil gelatinase-associated lipocalin [5, 11], estimation of NLR is more convenient and cheaper. Moreover, all hospitalized patients are routinely tested for NLR status. Our findings may assist medical staff to stratify patients based on risk, which may further assist in the implementation of prophylactic strategies and optimization of effective use of existing resources. Clinical outcomes of the patients could be significantly improved using this approach.

Our study identified other factors that exhibited independent correlation with the progression of postoperative AKI and in-hospital mortality, including sex, age, surgery type (neurosurgery, gastrointestinal surgery, other surgery), emergency surgery, ASA PS, *γ*-glutamyl transpeptidase, duration of MAP < 50 mmHg (0 min; 1–30 min; > 30 min) and urine output. Perioperative management (e.g., prevention of hypothermia; hemodynamic optimization; and avoiding nephrotoxins) may reduce postoperative adverse events in patients with these high-risk factors.

### Strengths and limitations

As per our understanding, this is the first study to demonstrate the association of preoperative NLR with postoperative AKI. Our findings could be of great clinical significance for preoperative NLR management in postoperative patients. This study included patients who underwent noncardiac surgeries in the largest tertiary hospital in China’s western region. Selection bias was reduced to a minimum using stringent inclusion criteria. The results of the study were further validated by PS matching and multivariable regression analysis on NLR. NLR was considered as a continuous variable to establish the potential linear relationship between NLR and postoperative AKI and mortality. An additional subgroup analysis was also carried out.

However, there are a few limitations to this study. Firstly, this study was carried out in a single center, which may not be a good representation of the clinical conditions. Secondly, there was unavailability of a few crucial pieces of information (such as postoperative urine output and preoperative information regarding drug use). The unavailability of this information could impact further assessment of the characteristics and pathogenesis of postoperative AKI. Thirdly, although we used propensity-score matching methods and multivariable logistic regression to reduce the impact of confounding variables, confounding bias could still be introduced due to unadjusted factors, such as the bispectral index of electroencephalogram (EEG) and perioperative care.

## Conclusion

To summarize, an elevated level of preoperative serum NLR is associated with an increased risk of postoperative AKI and in-hospital mortality in noncardiac surgeries. Our results suggest that NLR could be a potential biomarker that could be used in perioperative risk assessment of patients undergoing noncardiac surgeries.
